# Psychometric validation of the death literacy index and benchmarking of death literacy level in a representative uk population sample

**DOI:** 10.1186/s12904-022-01032-0

**Published:** 2022-08-13

**Authors:** Lisa Graham-Wisener, Paul Toner, Rosemary Leonard, Jenny M. Groarke

**Affiliations:** 1 Centre for Improving Health-Related Quality of Life, School of Psychology, Queen’s University Belfast, Belfast, BT7 1NN UK; 2 School of Social Sciences and Psychology, Penrith Kingswood Campus, Western Sydney University Locked Bag 1797, Penrith, NSW 2751 Australia; 3 School of Psychology, National University of Ireland Galway, University Road, Galway, Ireland

**Keywords:** Death literacy, Death literacy index, Public health, Palliative care, End of life care, Carers, Community development, Death education, Validation, UK

## Abstract

**Background:**

Death literacy includes the knowledge and skills that people need to gain access to, understand, and make informed choices about end of life and death care options. The Death Literacy Index (DLI) can be used to determine levels of death literacy across multiple contexts, including at a community/national level, and to evaluate the outcome of public health interventions. As the first measure of death literacy, the DLI has potential to significantly advance public health approaches to palliative care. The current study aimed to provide the first assessment of the psychometric properties of the DLI in the UK, alongside population-level benchmarks.

**Methods:**

A large nationally representative sample of 399 participants, stratified by age, gender and ethnicity, were prospectively recruited via an online panel. The factor structure of the 29-item DLI was investigated using confirmatory factor analysis. Internal consistency of subscales was assessed alongside interpretability. Hypothesised associations with theoretically related/unrelated constructs were examined to assess convergent and discriminant validity. Descriptive statistics were used to provide scaled mean scores on the DLI.

**Results:**

Confirmatory factor analysis supported the original higher-order 8 factor structure, with the best fitting model including one substituted item developed specifically for UK respondents. The subscales reported high internal consistency. Good convergent and discriminant validity was evidenced in relation to objective knowledge of the death system, death competency, actions relating to death and dying in the community and loneliness. Good known-groups validity was achieved with respondents with professional/lived experience of end-of-life care reporting higher levels of death literacy. There was little socio-demographic variability in DLI scores. Scaled population-level mean scores were near the mid-point of DLI subscale/total, with comparatively high levels of experiential knowledge and the ability to talk about death and dying.

**Conclusions:**

Psychometric evaluations suggest the DLI is a reliable and valid measure of death literacy for use in the UK, with population level benchmarks suggesting the UK population could strengthen capacity in factual knowledge and accessing help. International validation of the DLI represents a significant advancement in outcome measurement for public health approaches to palliative care.

**Pre-registration:**

https://osf.io/fwxkh/

## Background

The global death rate and demand for palliative care is projected to increase substantially over the next two decades [1], with an estimated 42 per cent increase in demand for palliative care in the UK by 2040 [2]. The ‘new public health approach’ to end-of-life care (EoLC) is concerned with the potential for increased scarcity of statutory palliative care provision as demand rises [3], but also questions the value of a model of care focused solely on institutionalised services, underpinned by the Biomedical Model. Public health approaches such as the Health Promoting Palliative Care model or ‘Compassionate Communities’ [4], advocate for a shift towards a social model of EoLC, where each social actor is empowered to contribute [3]. A core principle of new public health approaches to EoLC are around fostering community participation and agency with recognition of the substantial burden of informal carers in providing EoLC and the need for entire communities rather than professional service providers to support individuals at the end-of-life [5, 6].

Extensive qualitative research with individuals with lived experience of caring for someone dying at home by researchers in Australia [7–9] suggested that over time those in informal caring networks develop skills and abilities for providing EoLC. The capacity which is developed by individuals has been termed ‘death literacy’ and is defined by the authors as; ‘*the knowledge and skills that people need to make it possible to gain access to, understand, and make informed choices about end of life and death care options. People and communities with high levels of death literacy have context specific knowledge about the death system and the ability to put that knowledge into practice*’ [10]. Four theoretical facets of death literacy are proposed, described as knowledge, skills, experiential learning, and social action [11].

Although there is indication of a range of community-based new public health EoLC initiatives in practice, few are formally evaluated [12] which means there is little available evidence on the impact of such an approach. One identified challenge to evaluating community-based initiatives is the lack of an outcome measure which meaningfully captures the multi-dimensional impact of ‘Compassionate Communities’ intervention [12]. Existing tools largely measure individual constructs such as clinical concerns or knowledge, and do not include a focus on community support [13].

The recently developed Death Literacy Index (DLI; [13]) addresses this important gap. This is a 29-item measure designed to assess levels of death literacy across multiple contexts, including at a community/national level, and to evaluate the outcome of public health interventions. The development of the DLI was informed by an existing theoretical conceptualisation of death literacy [11] and relevant measures, and was refined with input from professionals with experience in the EoLC sector. The measure has previously been validated by the original authors [10, 13] who administered the measure to 1200 participants from the general population in Australia, with analysis involving exploratory and confirmatory factor analyses. This confirmed a structure with four subscales, two of which have two subscales. The DLI subscales reported high reliability and good internal consistency. Convergent validity was evidenced between scores on the DLI and items measuring objective knowledge of the death system, end-of-life actions and attitudes, and with a measure of death competence (Coping with Death Scale; [14]). The measure has also been piloted in several Australian community samples [10], and in one UK community sample (St Nicholas Hospice).

The DLI is the first rigorously developed measure of the construct of death literacy, which is a key outcome for public health interventions in palliative care (a priority public health area). Although the measure evidences good psychometric properties in an Australian context, it has not been validated in other international contexts so it is unclear how it performs cross-culturally. The current study will provide the first international validation of the DLI, in a representative UK population sample, and a benchmark of DLI and subscale scores for the UK. If the measure performs well, this will allow UK researchers, practitioners, and policymakers to evaluate community/organisation and national level strategies and interventions to increase death literacy.

### Aim

The primary aim of this study was to examine the psychometric properties of the Death Literacy Index (DLI, Version 1.0) in a UK population-level sample. The secondary aim was to provide a benchmark of DLI and subscale scores for the UK, and to examine demographic variability in scores.

The objectives were:

I. To determine the psychometric properties of the DLI in a UK population level sample, in relation to structural, construct validity, internal consistency, and interpretability
II. To provide a benchmark (scaled mean score) on the DLI and subscales in a UK population-level sample
III. To examine the demographic variability in the DLI in a UK population-level sample

## Methods

### Study design

A cross-sectional online survey, with validation of the Death Literacy Index informed and reported according to the COSMIN Study Design checklist for patient-reported outcome measurement instruments [15]. The study protocol was pre-registered on the Open Science Framework (https://osf.io/fwxkh/).

### Population and settings

Participants were prospectively recruited via an online crowdsourcing platform managed by Prolific Academic Ltd (http://www.prolific.co). A nationally representative sample of participants representing the target population was recruited from the estimated 41,000 UK residents on the panel, stratified across age, sex and ethnicity in alignment with the proportions reported in the UK Office of National Statistics Census data [16]. Prolific establishes the population strata, with a predetermined number of open slots into which eligible participants in the panel can enrol on a first-come basis. Inclusion criteria included: adults (≥ 18 years of age) currently living in the UK, and with capacity to express their opinion. Participants read a participant information sheet and provided explicit informed consent before completing the survey via Qualtrics online platform [17]. Responses were collected between 19^th^ October and 3^rd^ November 2020. Median completion time was approximately 10 min. A small financial incentive was offered for completion, equivalent to £9.51/hour.

### Measures

Measures included the Death Literacy Index (DLI; [13]) alongside several measures to assess construct validity. Death literacy was expected to be positively associated with death competency, with the Coping with Death Scale [14] included to assess convergent validity, alongside items to assess i) objective knowledge and ii) actions regarding discussion of death and dying. A negative association was expected between death literacy and loneliness, with the Short Revised UCLA Loneliness Scale [18] included to assess discriminant validity. Lastly, information on socio-demographic characteristics were collected, including individual experiences of death, dying and loss (e.g. working, volunteering or lived experience) to assess known group validity.

#### The death literacy index (DLI, version 1.0; [13])

A 29-item self-report measure of the construct of death literacy, with a higher-order factor structure composed of four subscales, two of which have two subscales; 1. Practical Knowledge (8 items) including the (i) ‘Talking Support’ subscale (4 items) and (ii) ‘Doing hands on care’ subscale (4 items), 2. Experiential Knowledge (5 items), 3. Factual Knowledge (7 items) and 4. Community Knowledge (9 items) including (i) ‘Accessing Help’ subscale (5 items) and (ii) ‘Support Groups’ subscale (4 items). Responses are on a 5-point Likert scale (from 1 to 5). Subscale scores are computed by summing items and scaling per number of items in subscale (with a range of scores between 0 and 10). Emerging evidence on the psychometric properties of the DLI in a community-based population in Australia is good [13], confirming structural, cross-cultural and construct validity, internal consistency, and interpretability. The measure has also been piloted in one UK community sample (Mildenhall, England as facilitated by St Nicholas Hospice). Leonard and colleagues in correspondence confirmed that in the UK community sample there were no items which participants found difficult or omitted. The scaled mean scores on the subscales/DLI total score ranged from 4.6- 7.5 with evidence of ceiling effects, and with good internal consistency (Cronbach’s alpha for the scale was 0.927 and sub-scales ranged from 0.794 to 0.904).

#### Coping with death scale [14]

A 30-item self-report measure of the construct of death competency. The scale assesses both one’s sense of competence in handling death and concrete knowledge concerning preparation for death. Participants are instructed to indicate the extent to which they agree with 30 statements using a 7-point Likert scale. Items are summed, with a range of scores between 30 and 210. The scale has shown good internal consistency and stability with various samples, as well as some evidence of construct validity in distinguishing hospice volunteers from controls and predicting death preparation behaviours [19]. Cronbach’s alpha in the current sample indicates good internal consistency (30 items, α = 0.94).

#### Short revised UCLA loneliness scale [18]

A 3-item self-report measure of the construct of loneliness. The scale measures three different aspects of loneliness, (social connectedness, relational connectedness, and self-perceived connectedness). Participants are instructed to indicate how often they feel that way with three statements, using a 3-point Likert scale (from 1 to 3). The items are summed. This is a widely used measure of loneliness, developed for large online surveys, and demonstrates good psychometric properties in relation to the full UCLA scale [20]. Good internal consistency (3 items, α = 0.86) was reported for the current sample.

#### Objective knowledge items

Developed by the original DLI authors [13], this includes four items to measure the objective knowledge of the death system. An example includes ‘What is palliative care?’ (response options; Care received only by people in the last few weeks or days of life, Care for people aged over 85, Care that aims to improve the quality of life of people with a life-threatening illness). Participants provide categorical answers, and correct items are summed.

#### Actions regarding discussion of death & dying items

Developed by the original DLI authors [13], this includes two items to measure the attitudes and actions to discussion of death and dying. The items are ‘In my community we discuss death and dying’ and ‘In my family we discuss death and dying’. Participants provide answers using a 5-point Likert scale (from 1 to 5).

## Data analysis

### Sample size calculation

The sample size estimation was calculated on the basis of the factor analysis. Where factor structure is known a sample size of > 200 is recommended [21]. A sample size of *n* = 399 meets multiple criteria, with some researchers recommending a sample size of at least 300 [22, 23] and others recommending participant to item ratios ranging from 5 to 10 participants per item [24], with any less than 3 participants per item deemed inadequate [25].

### Ethics

Research ethics approval was provided by the Queen’s University Belfast Engineering and Physical Sciences Faculty Research Ethics Committee (Reference; EPS 20_218) on 11^th^ September 2020. The study was conducted in accordance with the Declaration of Helsinki [26] and participants completed an informed consent statement prior to completion of the survey.

### Analysis

Data were exported from Qualtrics [17], and analysed using the Statistical Package for Social Science for Windows, Version 25 (SPSS Inc., Chicago, IL, USA), an alpha level of p < 0.05 was considered statistically significant. The ordinal responses of the DLI were treated as continuous data. There were no missing data as forced responses were used in the survey. The scaled mean of the subscales is used throughout as recommended by the measure’s authors for benchmarking of population level scores, with raw scores used for assessment of interpretability.

#### Objective 1

The psychometric properties of the DLI were evaluated according to standard methodology as outlined by COSMIN [15, 27].

##### Dimensionality

The validity of the factor structure identified in the original scale development study [13] was examined in the current study by confirmatory factor analysis (CFA) using Structural Equation Modelling (SEM) in Amos version 23 (SPSS Inc., Chicago, IL, USA). Preliminary analysis to confirm the suitability of the data for factor analysis included inspecting the correlation matrix for at least several moderate-strong inter-item correlations (> 0.3) and for no perfect multicollinearity (< 0.9). Sampling adequacy was also assessed by the Kaiser–Meyer–Olkin (KMO) value (threshold > 0.6) and Barlett’s Test of Sphericity (significance at < 0.05). Preliminary analyses evidenced sampling adequacy for factor analysis with largely moderate inter-item correlations but no perfect multicollinearity with all inter-item correlations < 0.83. A KMO value of 0.92 and a significant Barlett’s Test of Sphericity, χ2 (435) = 8150.66, p < 0.001 indicated suitability for factor analysis. Variance–covariance matrix with maximum likelihood (ML) estimation procedure was used for SEM, which is appropriate if there are more than three ordinal categories [28]. Assumptions for ML include multivariate normality. The univariate normality of the variables was assessed by kurtosis and skewness values, with recommended thresholds of moderate non-normality of < 2 for kurtosis and < 8 for skewness [29]. All the univariate skewness and kurtosis values were smaller than the recommended thresholds of moderate non-normality. At the multivariate level, multivariate kurtosis = 148.37 with a significant Mardia’s coefficient of 34.95, with threshold of < 5 indicating multivariate normality [30]. This suggested univariate normality and a multivariate departure from normality. The data was inspected for multivariate outliers by Mahalanobis distance value. Removing five true outliers (substantial distances from other cases) reduced the multivariate kurtosis to 127.20 and Mardia coefficient to 29.772. In all subsequent analyses, 394 participants are the focus. The initial model specified was the 29 items of the DLI, loading onto a hierarchical structure with 8 factors. A second model with a new item developed for the UK context (under Factual Knowledge scale) was tested, as specified a priori in the study pre-registration. This item asks about the contribution of ‘funeral home staff’, in place of an item referring to the contribution of ‘cemetery staff’.

Model fit was assessed using a series of indices, according to best practice [31]. A non-significant chi square goodness of fit test is indicative of a well-fitting model and was considered but is sensitive to sample size [28]. Additional model fit indices used are the normed chi square (Q), the comparative fit index (CFI), the root mean square of approximation (RMSEA), and the standardised root mean square residual (SRMR). Cut-offs of fit indices include; Q; acceptable criteria vary from under 2 [32] to less than 5 [33]; CFI: ≥ 0.90 and 0.95 reflect acceptable and excellent fit to the data, respectively [34]. RMSEA and SRMR; values between 0.05 and 0.09 indicating adequate model fit and values < 0.05 indicating a very good fit [35]. Modification indices available in CFA have been used to identify misspecification in the model. Decisions regarding modifications were based on theoretical in addition to psychometric considerations of item and scale content. We planned to eliminate items if they had low factor loadings (i.e., standardized regression coefficients) (< 0.40), or if modification indices suggested they had significant loadings (> 0.30) with unintended latent factors [28].

##### Internal consistency

After determining dimensionality based on theoretical assumptions and model fit according to standard criteria outlined above, items were evaluated for their psychometric properties. This involved examining the reliability of the unidimensional subscales separately by Cronbach's alpha and coefficient omega. Item to total correlations (r > 0.30 as a minimum criterion [36]. A Cronbach’s alpha coefficient between 0.70 and 0.95 indicates good internal consistency without homogeneity [37].

##### Construct validity

Is the extent to which scores on an instrument relate to other measures (convergent validity/discriminant validity) or produce expected differences in scores between ‘known’ groups (known-groups validity). It is given a positive rating if at least 75% of the results are consistent with predefined hypotheses. Construct validity of the DLI was tested against items measuring people’s knowledge of the death system, a measure of death competence and for respondents identifying as having professional or lived experience of death, dying and loss. Pearson’s correlation coefficients or ANOVA were undertaken according to predefined hypotheses of convergent/discriminant validity. We define the strength of the correlation as strong (0.7–1.0), moderate (0.4–0.7), weak (0.2–0.4) and absent (0.0–0.2) [38]. We define the strength of the ANOVA as small (Eta sq = 0.01), medium (0.06) or large (0.14) [38].

##### Convergent validity

H1: Moderate positive association expected between an individual’s objective knowledge of the death system and the DLI and subscale scores.

H2: Moderate positive association expected between items of individual’s scores on the Coping with Death Scale [14] and the DLI and subscale scores.

H3: Moderate positive association expected between items of individual’s actions in relation to discussing death and dying and the DLI and subscale scores.

##### Known-groups validity

H4: Moderate positive association expected for individuals with experience working/volunteering or with prior lived experience of death, dying and loss and the DLI and subscale scores.

##### Discriminant validity

H5: Moderate negative associations expected between items of individual’s scores on the Short Revised UCLA Loneliness Scale [18] and the DLI and subscale scores.

##### Interpretability

Was determined by analysing the distribution of participants’ total scores (median, range, interquartile range), with floor and ceiling effect indicated if 15% of respondents achieved the lowest or highest possible score, respectively.

##### Objectives 2 & 3:

Descriptive statistics were used to provide a scaled mean score on the DLI and subscales. ANOVA were used to examine the relationship between demographic variables and DLI/subscale scores.

## Results

There were 417 responses to the survey. Responses were screened for data quality including for potential duplicate responses and lack of engagement, with 18 responses removed for incomplete data or having a completion time less than half the median completion time. Responses were forced, so there were no missing data. After inspecting the included data (*n* = 399) for multivariate normality, five outliers were removed. The included sample (*n* = 394) were a mean age of 45.8 years old (SD 15.73). The majority of participants reported to not have any personal or professional end-of-life care experience (*n* = 243, 61.7%). A minority reported to have personal end-of-life care experience, considering themselves (*n* = 10, 2.5%) or a close person (*n* = 37, 9.4%) to be in the last few years of life, or reporting to have been bereaved in the last two years (*n* = 67, 17%). A minority reported to have professional end-of-life experience, either working or volunteering with people at end of life (*n* = 41, 10.4%) or individuals experiencing grief or bereavement (*n* = 27, 6.9%) or having attended training on helping people with dying, grief or bereavement (*n* = 29, 7.4%). Table 1 shows the other medical and socio-demographic information for this sample.

**Table 1:** Medical and socio-demographic characteristics of sample (*n* = 394)

	**N**	**%**
**Gender**		
Male	193	49.0
Female	200	50.8
Other	1	0.3
**Ethnicity**		
White	313	79.4
Asian ethnic group	38	9.6
African ethnic group	19	4.8
Arab ethnic group	2	0.5
Latino or Hispanic ethnic group	2	0.5
Other	9	2.3
Mixed/multiple	11	2.8
**Language spoken at home**		
English	354	89.8
Mainly English	23	5.8
Other language	17	4.3
**Relationship Status**		
Single	87	22.1
Partnered but not living together	33	8.4
Married or living with a partner	239	60.7
Divorced	26	6.6
Separated but not divorced	5	1.3
Widowed	3	0.8
Other	1	0.3
**Highest Level of Education**		
Lower secondary level	48	12.2
Upper secondary level	84	21.3
Post-secondary non-tertiary general education	59	15.0
Undergraduate degree	121	30.7
Postgraduate qualification	71	18.0
Doctoral degree	7	1.8
Other	4	1.0
**Employment Status**		
Employed full-time	162	41.1
Employed part-time	60	15.2
Casual	11	2.8
Not working	31	7.9
Retired	57	14.5
Actively seeking work	18	4.6
Student	31	7.9
Other	24	6.1
**Annual household income (pre-tax)**		
< £12,500	53	13.5
£12,501 to £50,000	239	60.7
£50,001 to £150,000	96	24.4
Over £150,000	6	1.5
**Dependents**		
Children	208	52.8
Dependent adults	44	11.2
**Religious or spiritual background**		
Yes	116	29.4
No	278	70.6
**Belief in an afterlife**		
Yes	98	24.9
No	133	33.8
I don’t know or am unsure	163	41.4
**Location**		
Rural- isolated dwelling, hamlet or village	71	18.0
Town- small or large town	196	49.7
City	127	32.2
**Chronic Health Conditions**		
Chronic Physical Illness	66	16.8
Chronic Mental Illness	22	5.6
Terminal Illness	1	0.3

### Dimensionality

ML estimation method with bootstrapping was used to provide a more accurate estimation of standard errors in relation to p values and confidence intervals. The Bollen-Stine bootstrap p was used as an alternative to χ2 [39]. The bootstrapping sample was 250, with 95% confidence interval as recommended by Nevitt and Hancock [40].

**Fig. 1:**
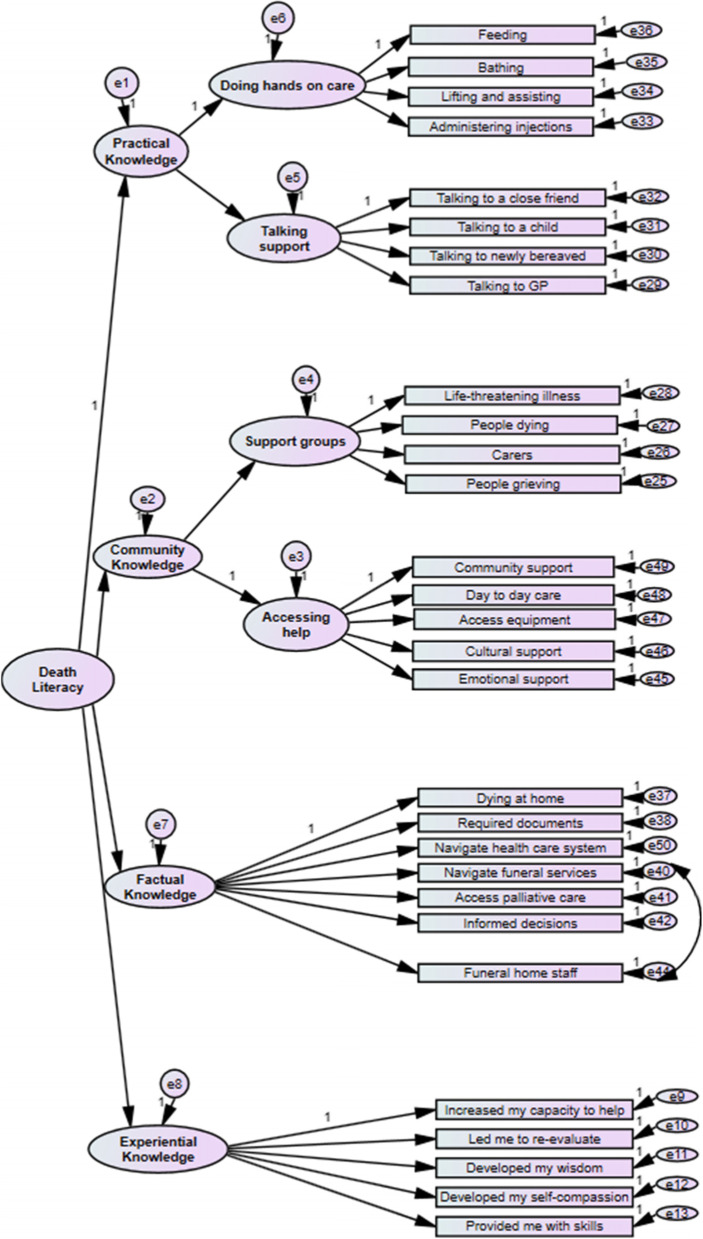
Path diagram of DLI final model

The first model specified was the 29 items loading on to their 8 respective factors as per the original model reported in the initial development of the DLI [13]. This refers to 4 subscales, two of which have their own 2 subscales; 1. Practical Knowledge including the (i) ‘Talking Support’ subscale and (ii) ‘Doing hands on care’ subscale, 2. Experiential Knowledge, 3. Factual Knowledge and 4. Community Knowledge including (i) ‘Accessing Help’ subscale and (ii) ‘Support Groups’ subscale. This model was a good fit of the data; χ^2^ (369) = 822.12, *p* < 0.001, Bollen-Stine bootstrap *p* = 0.004, Q = 2.23, CFI = 0.94, RMSEA = 0.07 (90% CI, 0.050-0.061), SRMR = 0.07. There were no items with low factor loadings (< 0.40), and no modification indices suggesting significant cross-loadings (> 0.30). A second model was specified to test whether the inclusion of a new item in the Factual Knowledge subscale (‘*I know the contribution the funeral home staff can make at end of life*’) impacted model fit. This replaced an original item (‘*I know about the contribution the cemetery staff can make at end of life’*) as it was deemed more culturally appropriate for UK respondents. There was a slight reduction in terms of the model fit for this second model but this model was still a good fit on the majority of indices; χ^2^ (369) = 871.69, *p* < 0.001, Bollen-Stine bootstrap *p* = 0.004, Q = 2.36, CFI = 0.93, RMSEA = 0.07 (90% CI, 0.054-0.064), SRMR = 0.07. Nonetheless, the factor loading of the new item (Q24) was greater (0.71) than the original item (0.63), with the reliability and factor loading of the Factual Knowledge subscale on the death literacy latent variable remaining largely consistent. Modification indices, however, showed a degree of variance shared between the new item and another item on the same subscale (‘*I know how to navigate funeral services and options*’). In a third model, the new replacement item was retained (‘*I know about the contribution the cemetery staff can make at end of life’*) and its error term was co-varied with the item (‘*I know how to navigate funeral services and options*’). This resulted in overall model fit indices superior to the initial specified model; χ^2^ (368) = 812.83,* p* < 0.001, Bollen-Stine bootstrap *p* = 0.004, Q = 0.2.21, CFI = 0.94, RMSEA = 0.07 (90% CI, 0.050-0.061), SRMR = 0.07. The path diagram for this final model is presented in Fig. 1. The final 29 items of the DLI measure validated for UK context, their beta weights (β), that is their factor loadings, as well as, the proportion of variance in the latent construct explained by that item (r^2^) are reported in Table 2.

**Table 2:** The Death Literacy Index, internal consistency, and descriptive statistics of 8 subscales, and psychometric properties of 29 final scale items

Subscales and items	β (95% CI)	*p*	*r*^*2*^	α/ ω	M (SD)^1^
**Practical Knowledge**	0.824 (0.676, 0.949)	.020	.68	.791/.784	5.35 (1.91)
**Doing hands on care**	0.698 (0.595, 0.850)	.005	.49	.763/.776	4.73 (2.43)
Q1. Feeding a person or assisting them to eat	0.856 (0.807, 0.907)	.006	.73		
Q2. Bathing a person	0.848 (0.797, 0.910)	.006	.72		
Q3. Lifting a person or assisting to transfer them	0.598 (0.511, 0.680)	.008	.36		
Q4. Administering injections	0.415 (0.320, 0.517)	.005	.17		
**Talking support** Q5. Talk about death, dying or grieving to a close friend	0.679 (0.552, 0.823) 0.705 (0.618, 0.782)	.008 .006	.46 .50	.780/.784	5.96 (2.16)
Q6. Talk about death, dying or grieving to a child	0.585 (0.494, 0.675)	.011	.34		
Q7. Talk to a newly bereaved person about their loss	0.677 (0.594, 0.740)	0.12	.46		
Q8. Talk to a GP about support at home or in their place of care for a dying person	0.788 (0.714, 0.840)	.005	.61		
**Community Knowledge**	0.863 (0.764, 0.948)	.008	.74	.922/.922	4.48 (2.29)
**Support groups** Q9. People with life threatening illnesses	0.608 (0.514, 0.683) 0.883 (0.843, 0.913)	.022 .015	.37 .78	.923/.923	5.06 (2.43)
Q10. People who are dying	0.935 (0.903, 0.954)	.012	.87		
Q11. Carers for people who are dying	0.847 (0.790, 0.879)	.021	.72		
Q12. People who are grieving	0.801 (0.739, 0.846)	.011	.64		
**Accessing help** Q13. Access community support	0.965 (0.886, 1.073) 0.851 (0.799, 0.885)	.005 .013	.93 .73	.927/.928	3.91 (2.77)
Q14. Provide day to day care for the dying person	0.889 (0.852, 0.917)	.006	.79		
Q15. Access equipment required for care	0.897 (0.862, 0.929)	.006	.81		
Q16. Access culturally appropriate support	0.853 (0.816, 0.891)	.009	.73		
Q17. Access emotional support for myself	0.751 (0.670, 0.804)	.021	.56		
**Factual knowledge** Q18. I know the law regarding dying at home	0.809 (0.721, 0.872) 0.711 (0.641, 0.783)	.004 .005	.65 .51	.924/.925	3.05 (2.60)
Q19. I feel confident in knowing what documents you need to complete in planning for death	0.835 (0.789, 0.873)	.012	.70		
Q20. I know how to navigate the health care system to support a dying person to receive care	0.907 (0.870, 0.935)	.010	.82		
Q21. I know how to navigate funeral services and options	0.741 (0.671, 0.791)	.013	.55		
Q22. I know how to access palliative care in my area	0.872 (0.838, 0.897)	.011	.76		
Q23. I have sufficient understanding of illness trajectories to make informed decisions around medical treatments available and how that will shape quality of end of life	0.786 (0.740, 0.831)	.003	..62		
Q24. I know about the contribution the funeral home staff can make at end of life	0.707 (0.650, 0.771)	.008	.50		
**Experiential Knowledge** Q25. Increased my emotional strength to help others with death and dying processes	0.623 (0.467, 0.743) 0.710 (0.634, 0.780)	.014 .008	.39 .50	.868/.871	6.26 (2.12)
Q26. Led me to re-evaluate what is important and not important in life	0.676 (0.575, 0.746)	.006	.46		
Q27. Developed my wisdom and understanding	0.843 (0.776, 0.887)	.013	.71		
Q28. Made me more compassionate toward myself	0.731 (0.654, 0.785)	.008	.53		
Q29. Provided me with skills and strategies when facing similar challenges in the future	0.829 (0.769, 0.872)	.011	.69		

^1^Range is from 0–10, *β* standardised regression coefficient. *r*^*2*^ squared regression coefficient. *CI* bootstrapped confidence interval. *Α* Cronbach’s alpha

*Ω* Coefficient omega

### Internal consistency

The Cronbach’s alpha for each subscale were between α = 0.76 and α = 0.93, with the Omega coefficient between ω = 0.78 and ω = 0.93 (see Table 2), evidencing good internal consistency without homogeneity. All item to total correlations met the minimum criteria of r > 0.30.

### Construct validity

#### Convergent validity

Convergent validity can be evidenced with significant moderate positive associations between the subscales/DLI total score and objective knowledge of the death system, between the DLI and death competence (Coping with Death Scale; [14]), and between the DLI and actions relating to death and dying in the family and community (see Table 3) as hypothesised. Overall, more than 75% of the results are consistent with the predefined hypotheses in terms of direction of the effect (H1, H2 & H3). However, the strength of the correlation was not as expected and was weak for the subscales/DLI total score for the majority of constructs, apart from death competency where moderate correlations as hypothesised were observed.

**Table 3:** Convergent validity and discriminant validity of the Death Literacy Index (r)

Subscales	Objective knowledge of death system	Coping with Death Scale	Actions relating to discussing death and dying – community	Actions relating to discussing death and dying- family	UCLA Loneliness Scale
**Practical Knowledge**	.197**	.631**	.342**	.417**	-.104*
Doing hands on care	.134**	.403**	.242**	.257**	-.038
Talking support	.195**	.657**	.331**	.445**	-.141**
**Community Knowledge**	.209**	.538**	.348**	.302**	-.165**
Support groups	.167**	.416**	.292**	.237**	-.166**
Accessing help	.199**	.525**	.321**	.292**	-.128**
**Factual knowledge**	.234**	.630**	.247**	.305**	-.122**
**Experiential Knowledge**	.132**	.520**	.302**	.401**	-.050
**DLI Total**	.251**	.746**	.394**	.451**	-.144**

Correlation is significant at the 0.05 level (1-tailed).*, Correlation is significant at the 0.01 level (1-tailed).**

#### Known groups validity

Known groups validity was assessed for individuals identifying as having professional expertise in end-of-life care or bereavement, professional training, or lived experience. Due to a low number of participants identifying as being in the last years of life (*n* = 10), this subgroup was not assessed. Table 4 shows that all roles, apart from being a carer of someone who is at the end of life, are related to higher mean scores on all the DLI subscales in comparison to individuals identifying with none of the ‘expert’ roles in line with hypothesised findings (H4). The eta-square statistics show that the strength of these relationships was either medium to large on the subscales, and large for the DLI total score. Individuals identifying as a carer of someone at the end of life report significantly higher levels of death literacy on the majority of subscales and the DLI total score, however all effect sizes were small.

**Table 4:** Known-groups validity of the death literacy index

Subscales	I work or have worked with people at end of life, including volunteering (*n* = 41)			I work or have worked in a job where I support/ed people through grief and loss, including volunteering (*n* = 27)			I have attended training on helping people with dying, grief or bereavement (*n* = 29)			I am carer/family member/partner/spouse/friend of someone who is thought to be in the last few years of their life (*n* = 37)			I am a bereaved carer/family member/partner/spouse/friend of someone who has died in the last 2 years (*n* = 67)		
	**Mean**^1^ **(SD)**	**Welch’s F statistic and sig level**	**Eta sq**	**Mean** **(SD)**	**Welch’s F statistic and sig level**	**Eta sq**	**Mean** **(SD)**	**Welch’s F statistic and sig level**	**Eta sq**	**Mean** **(SD)**	**Welch’s F statistic and sig level**	**Eta sq**	**Mean** **(SD)**	**Welch’s F statistic and sig level**	**Eta sq**
**Practical Knowledge**	6.94 (1.67)	48.73***	0.130	6.55 (1.59)	24.27***	0.066	6.45 (1.78)	18.64***	0.061	5.88 (1.71)	9.45**	0.030	5.80 (1.86)	11.31**	0.036
Doing hands on care	6.59 (2.47)	29.78***	0.106	6.02 (2.45)	11.71**	0.046	5.71 (2.81)	6.49*	0.032	5.00 (2.25)	2.82	0.010	5.13 (2.39)	5.99*	0.020
Talking support	7.30 (1.67)	35.18***	0.080	7.08 (1.88)	15.82***	0.045	7.20 (1.64)	24.32***	0.055	6.76 (1.89)	12.71**	0.036	6.47 (2.18)	9.55**	0.030
**Community Knowledge**	6.45 (2.34)	40.91***	0.147	5.93 (2.33)	17.58***	0.073	6.55 (2.52)	28.18***	0.124	4.68 (2.37)	3.01	0.013	5.54 (2.29)	25.87***	0.086
Support groups	6.55 (2.64)	17.21***	0.070	6.69 (4.49)	15.19***	0.061	6.77 (2.81)	14.00**	0.068	5.05 (2.55)	0.49	0.002	5.74 (2.46)	8.92**	0.031
Accessing help	6.35 (2.64)	51.14***	0.164	5.17 (2.77)	12.62**	0.053	6.33 (2.86)	31.89***	0.128	4.31 (2.80)	5.28*	0.022	5.34 (2.76)	33.21***	0.108
**Factual Knowledge**	4.92 (3.15)	24.40***	0.128	4.13 (3.10)	8.01**	0.050	5.17 (2.91)	24.82***	0.127	3.64 (2.96)	6.04*	0.033	4.57 (2.93)	31.94***	0.127
**Experiential Knowledge**	7.43 (1.97)	28.97***	0.086	7.33 (1.92)	19.01***	0.058	7.62 (1.73)	32.94***	0.083	6.76 (1.77)	12.49**	0.034	7.19 (1.91)	33.74***	0.090
**DLI Total**	6.44 (1.83)	53.83***	0.203	5.98 (1.90)	21.52***	0.106	6.45 (1.88)	37.74***	0.167	5.24 (1.70)	11.69**	0.049	5.77 (1.81)	41.02***	0.143

^1^Range is from 0–10 * Significant at the *p* < 0.05 level, ** Significant at the *p* < 0.01 level, ***Significant at the *p* < 0.001 level. Eta Sq. interpreted as .01 “small”; .06 “medium”; .14 “large” (Cohen, 1988)

#### Discriminant validity

There was a significant negative association between the majority of the DLI subscales/DLI total score and loneliness (Short Revised UCLA Loneliness Scale; [18]) (see Table 3) in line with what was predicted (H5). However, the eta-square statistics show the strength of these relationships were weak overall and not the moderate associations expected.

#### Interpretability

Interpretability was assessed using the individual raw data for each subscale, i.e. the item totals of participants’ scores. The participant’s total score on each subscale represented the total possible range for all subscales (see Table 5). There was no evidence of floor or ceiling effects on DLI total score, or the majority of subscales except for ‘Factual Knowledge’. Using the criterion of > 15% of respondents achieving the lowest possible score, there is some evidence of a floor effect for this subscale.

**Table 5:** Median, range, interquartile range and floor and ceiling effects of the Death Literacy Index

Subscales	*Mdn*	Range	IQR	Floor & Ceiling effects
**Practical Knowledge**	25.0	8–40 (possible range is 8–40)	8.0	3 participants (0.3%) had the lowest possible total score, and 2 participants (0.5%) had the highest possible total score
Doing hands on care	12.0	4–20 (possible range is 4–20)	5.0	12 participants (3.0%) had the lowest possible total score, and 9 participants (2.3%) had the highest possible total score
Talking support	14.0	4–20 (possible range is 4–20)	4.0	5 participants (1.3%) had the lowest possible total score, and 16 participants (4.1%) had the highest possible total score
**Community Knowledge**	25.0	9–45 (possible range is 9–45)	13.0	13 participants (3.3%) had the lowest possible total score, and 5 participants (1.3%) had the highest possible score
Support groups	12.0	4–20 (possible range is 4–20)	6.0	19 participants (4.8%) had the lowest possible total score, and 17 participants (4.3%) had the highest possible total score
Accessing help	12.0	5–25 (possible range is 5–25)	9.0	57 participants (14.5%) had the lowest possible total score, and 8 participants (2%) had the highest possible total score
**Factual Knowledge**	14.0	7–35 (possible range is 7–35)	11.0	61 participants (15.5%) had the lowest possible total score, and 4 participants (1%) had the highest possible score
**Experiential Knowledge**	18.0	5–25 (possible range is 5–25)	5.0	5 participants (1.3%) had the lowest possible total score, and 17 participants (4.3%) had the highest possible total score
**DLI Total**	84.0	40–143 (possible range is 29–145)	29.0	No participants had the lowest or highest possible total score

### UK population DLI benchmarks

The scaled mean scores for each of the subscales and the DLI total score is reported for the UK population (see Table 6). Individuals from the UK appear to have high levels of experiential knowledge and the ability to talk about death and dying, relative to other subscales.

**Table 6:** Scaled mean scores for the UK on DLI and its subscales

Subscales	UK Population (*n* = 394)Scaled Mean
**Practical Knowing (TOTAL 8 items)**	5.35 (1.91)
Hands on support (4 items)	4.73 (2.43)
Talking support (4 items)	5.96 (2.16)
**Community Knowledege (TOTAL 9 items)**	4.48 (2.29)
Community support groups (4 items)	5.06 (2.43)
Accessing help (5 items)	3.91 (2.77)
**Factual Knowledge (7 items)**	3.05 (2.60)
**Experiential Knowledge (5 items)**	6.16 (2.12)
**DLI TOTAL**	4.76 (1.73)

^1^Range is from 0–10

### Relationship between DLI and demographic variables

In relation to demographic variability in the DLI, the majority of demographic variables were either non-significant or reported weak effect sizes (see Table 7), demonstrating little variability in DLI to be explained by demographics. The following demographic variables were not significantly associated with the DLI at the 0.05 significance level; gender, highest level of education, employment status, annual household income, relationship status, caring for dependent adults, having a chronic mental illness, and belief in an afterlife. Due to small subgroup size, associations could not be explored for individuals with terminal illness.

**Table 7:** Summary of significant relationships between demographic variables and the death literacy index

	**Direction of relationship**	**Welch F statistic and significance level**	**Eta Sqr**
Age	Positive	8.39***	0.071
Rural location	Positive	3.41*	0.017
Having children	Positive	13.14***	0.032
Chronic physical health condition	Positive	4.15*	0.012
Religious background	Positive	8.16**	0.022

^*^Significant at the *p* < 0.05 level, ** Significant at the *p* < 0.01 level, ***Significant at the *p* < 0.001 level. Eta Sq. interpreted as .01 “small”; .06 “medium”; .14 “large” (Cohen, 1988)

The eta-square statistic for age reports a moderate effect size. Post hoc analysis using the Games-Howell criterion for significance indicated a positive relationship with age, with the DLI mean score higher for > 58 year olds (M = 3.11, SD = 0.77) than in 38–47 year olds (M = 2.76, SD = 0.69) or 28–37 year olds (M = 2.61, SD = 0.52), and the DLI mean score higher in 48–57 year olds than 28–37 year olds. The relationship with age was however not linear, with 28–37 year olds reporting a lower DLI mean score than 18–27 year olds (M = 2.90, SD = 0.61).

## Discussion

This is the first study to validate the Death Literacy Index (DLI; [13]) in the UK, with evidence suggesting that the DLI is a reliable and valid measure of death literacy in this population. In addition to providing the psychometric evaluation needed for this measure to be used in the UK, this study is one of the first to validate the DLI in an international context. This suggests that the measure performs well outside of Australia where it was originally developed [13]. The authors are aware of ongoing efforts to validate the DLI in Sweden, Belgium and the Netherlands.

The original higher-order factor structure was a good fit for the UK data. Model fit was improved with the addition of a substituted item for the UK context on the contribution of ‘funeral home staff’ (replacing ‘cemetery staff’) which loaded more strongly on to the ‘factual knowledge’ subscale. The authors would therefore recommend use of this substituted item when administering the DLI in the UK. All other items loaded well on to their respective subscales. The lowest loading items referred to administering injections, lifting a person or assisting to transfer them, and talking to a child about bereavement. The item relating to administering injections would not be applicable across all EoLC situations and so may be expected to not explain a high degree of variance. The other two items not loading as strongly is more unexpected and may reflect a lack of direct involvement in EoLC within the sample. This is worthy of further investigation, and a cognitive interviewing study is being undertaken by the lead author to assess the content validity of the DLI in the UK.

The DLI subscales possess good reliability (i.e., internal consistency), with the original DLI authors suggesting that individual subscales could be used alone if reliable [13]. Interpretability is also good, however floor effects were observed on the ‘factual knowledge’ subscale. Indeed, this was the subscale with the lowest scaled mean score for the UK sample. However, as the floor effects only just meet the threshold, this is unlikely to be a major cause for concern, with the DLI capable of measuring high and low death literacy. The DLI is also valid having demonstrated the expected positive and negative associations with related constructs, evidencing convergent and discriminant validity. Reassuringly, the DLI was moderately associated with the Coping with Death Scale [14], demonstrating that death literacy and death competency are related but distinct constructs. Effect sizes for the correlations with objective knowledge of the death system and actions regarding death and dying were smaller than expected and may reflect measurement error as validated measures were not used in order to restrict survey length. Although it does not measure understanding of the death system as a whole, future validation studies may consider using the Palliative Care Knowledge Scale (PaCKS; [41]) to assess objective knowledge. Effect sizes for the negative correlations between the DLI and loneliness were also smaller than expected. A consideration is that a construct such as perceived functional social support may be expected to be more highly correlated with death literacy than loneliness and could be explored in future research.

Known groups validity was demonstrated with individuals with professional or lived experience of EoLC reporting higher levels of death literacy as expected. However, for the subgroup identifying as a ‘carer/family member/partner/spouse/friend of someone who is thought to be in the last few years of their life’ scores were not higher on all of the DLI subscales. This may be due to how this group were defined, introducing significant heterogeneity. For example, the group may reflect individuals who are not directly involved in providing support for an individual at end-of-life. The group may also reflect individuals who are at the start of their caring journey, which raises an important question around when death literacy is developed along the caregiving trajectory. Using the DLI in research with carers could help inform our theoretical understanding of how and when death literacy develops, and the subsequent impact. There is increasing interest in the risk and protective factors for complicated grief [42], with greater preparedness for death, for example, shown to be a protective factor [43, 44]. With the DLI shown to be a valid and reliable measure of death literacy within the UK, there is an opportunity to develop robust evidence on how components of death literacy may improve end-of-life experiences both for individuals with life-limiting diagnoses and their close persons.

The current study provides, for the first time, UK population level benchmarks for the DLI total score and the various subscales. These benchmarks can be used to inform which components of death literacy may be most valuable to target at a population level through public health interventions and will be useful for researchers and practitioners to use as population baselines to compare scores within their own communities. Individuals from the UK appear to have, relative to other subscales, high levels of experiential knowledge and the ability to talk about death and dying. It must however be recognised that all population level benchmarks are near the mid-point of each subscale, and there is considerable opportunity to strengthen capacity in all areas of death literacy. For example, a recent survey in Northern Ireland [45] reported significant barriers to individuals talking about death and dying, such as fear of upsetting self or others and apprehension at navigating sensitive conversations. Key areas to strengthen capacity at a population level are around factual knowledge and accessing help. This is supported by recent UK research reporting a lack of familiarity with EoLC terminology and processes, and a lack of awareness on how to access support [46]. There is a lack of formally evaluated community-based EoLC interventions [12]. In addition to informing best value targets for novel interventions, the validation of the DLI in a UK context also provides a useful measure to evaluate the impact of such initiatives.

The population level benchmarks established in the current UK study are similar to the levels of death literacy reported in the Australian population [13]. However, the timing of both studies is a key contextual difference with the Australian data collected pre-pandemic, and the UK data mid-pandemic. Within the context of a mass-bereavement event, it is reasonable to assume that there would be greater opportunity for experiential learning, with the experiential knowledge subscale reporting the highest scaled mean score for the UK sample. This underscores the value of using the DLI to measure population trends in death literacy over time, with measurement of death literacy a key recommendation in a recent policy report [47]. It is an open-question as to whether the COVID-19 pandemic has contributed positively to communities’ capacity to provide EoLC, and indeed the extent to which death literacy can be sustained over time within communities. There is a desire from the general public to learn from those with professional and lived experience of EoLC [45], with the challenge being how to translate this into community-based interventions without increasing the recognised burden on informal carers.

As with the validation of the DLI in the original Australian sample [13], there was little socio-demographic variability in the current study implying the measure is applicable across social contexts. Although having a religious background and a chronic physical health condition report a significant relationship with higher levels of death literacy, the effect size is small. This is perhaps surprising given the opportunity to support individuals to develop death literacy in faith communities and health and social care settings. Only age reported a moderate effect size which may be expected, given that death literacy is suggested to develop from personal experience [10], with exposure to death, dying and loss accumulating over time. However, young adults have previously described experiencing exclusion from conversations relating to care decisions, serious illness and death, leading to a feeling of being ill-prepared [48]. This emphasises the importance of a life-course approach to death literacy, with respondents in our previous research suggesting that death literacy should be provided equal status to sexual health education in school settings [45]. The relationship between age and death literacy is not strictly linear in the current study, 28–37 year olds reporting a lower DLI mean score than 18–27 year olds, and there is a significant relationship between having children and higher levels of death literacy. Optimistically, this may reflect public health approaches to EoLC becoming more embedded for younger generations. The majority of research on public health approaches to EoLC has however focused on older adults [12] or solely on understanding of palliative and end of life care [48]. A more in-depth understanding of death literacy across the life-course would be a valuable focus for future research.

The current study has a number of strengths. The use of a population sample representative of age, gender and ethnicity provides confidence in the benchmarks, and addresses a limitation with the previous validation study [13], and the validation of death and dying measures more broadly [49]. The study followed best practice COSMIN guidelines [15] for assessment of structural, construct validity, internal consistency, and interpretability. However, the sample size in two subgroups for assessment of known groups validity was inadequate according to COSMIN recommendations. We also were not able to assess cross-cultural validity as planned in our pre-registered protocol, due to sample size within subgroups. Future research should focus on ascertaining the performance of the measure across different populations, in different age groups for example. The content validity of the measure was not assessed prior to the current study (the replacement item was developed by the research team), and ongoing research will address this important gap. The method of recruitment (via a panel) must also be considered, where self-selection of interested individuals may have led to an over-estimation of the levels of death literacy. The responsiveness of the DLI is still uncertain and given the potential use of the measure in evaluating public health interventions, this will be a priority to ascertain going forward. Future research with informal carers in particular is recommended, to ensure the measure performs well in this important context. Lastly, it must be recognised that the aim of this study was to establish the psychometric properties of the DLI at a population-level.

## Conclusion

The DLI is a valid and reliable measure of death literacy for use by researchers and practitioners in a UK context. Developing public health approaches to palliative care is a priority for the majority of palliative care service providers in the UK [50], yet the evidence base for public health approaches to palliative care is lacking with few formal evaluations [12]. The current study makes a novel contribution to these efforts by providing population-level benchmarks for the UK of the various components of death literacy to guide intervention development, and by evidencing the validity and reliability of the DLI as a measure of death literacy to be used to evaluate public health initiatives.

## Data Availability

The datasets used and/or analysed during the current study are available from the corresponding author on reasonable request. For a copy of the full Death Literacy Index measure, please contact Prof Rosemary Leonard (r.leonard@westernsydney.edu.au).

## Abbreviations

DLI: Death literacy index
EoLC: End of life care
